# Cold microwave plasma jets for wound healing: antimicrobial efficacy, mechanisms and changes in microbial cells

**DOI:** 10.1038/s41598-026-42650-5

**Published:** 2026-03-06

**Authors:** Kristína Trebulová, Veronika Loupová, Barbora Chobotská, Lukáš Kletzander, Přemysl Menčík, Zdenka Kozáková, Jan Hrudka, Joanna Pawlat, Pavel Kulich, František Krčma

**Affiliations:** 1 Faculty of Chemistry, Brno University of Technology, Purkyňova 118, 612 00 Brno, Czech Republic; 2 University of Chemistry and Technology Prague, Technická 5, Dejvice, 166 28 Praha 6, Czech Republic; 3 Veterinary Research Institute, Hudcova 296/70, 621 00 Brno, Czech Republic; 4 Department of Electrical Engineering and Smart Technologies, Lublin University of Technology, Nadbystrzycka Street 38A, 20-618 Lublin, Poland

**Keywords:** Plasma medicine, Oxidative stress response, RONS mapping, Plasma‑cell interaction, Electron microscopy, Biotechnology, Microbiology

## Abstract

Given the increasing prevalence of antibiotic-resistant microorganisms, alternative disinfection strategies are required. This study explores the antimicrobial potential of cold atmospheric plasma (CAP) as a non-thermal decontamination method for medical applications. The results confirm the efficacy of cold microwave plasma jets in the inactivation of *Staphylococcus epidermidis*,* Escherichia coli*,* Cutibacterium acnes*, and *Nakaseomyces glabratus*. Optimal treatment conditions ensuring both the antimicrobial efficacy and the safety for living tissue were established. Experiments in enclosed or open-air environment and the use of colorimetric agents confirmed that RONS, rather than UV radiation, are primarily responsible for microbial inactivation. Possible inhibition mechanisms induced by the CAP treatment were examined using scanning (SEM) and transmission (TEM) electron microscopy. The analyses revealed progressive morphological and intracellular changes in yeast cells following the plasma treatment, including localized thinning and perforation of the cell wall, vacuole enlargement, enhanced vesicle formation, protoplast aggregation and leakage of intracellular content.

**Supplementary Information:**

The online version contains supplementary material available at 10.1038/s41598-026-42650-5.

## Introduction

Wound healing is a complex biological process involving interactions between dermal and epidermal cells, the extracellular matrix, plasma proteins, and various fascial layers^1,2^. However, this process is frequently disrupted by bacterial infections, which arise from microbial proliferation in an open wound. In clinical practice, great emphasis is placed on both the treatment of early-stage infections and the prevention of infection onset. Nevertheless, the wound treatment remains challenging particularly due to biofilm formation and the growing problem of antimicrobial resistance. As a result of inefficient treatment, many wounds turn to their chronic stage^3,4^.To prevent and manage infections, antiseptics and antibiotics are widely used^5,6^. However, strong antiseptics can potentially delay the wound healing if they damage skin cells involved in the regenerative process^7^. The overuse and misuse of antibiotics have led to the emergence of antibiotic-resistant bacterial strains. Currently, it is estimated that approximately 70% of ifection‑causing bacteria exhibit resistance to at least one antibiotic^8–10^. This growing threat of antibiotic resistance has prompted increasing interest in new non‑chemical antimicrobial treatments^11^.

Cold atmospheric plasma (CAP) represents a promising alternative in infection control and wound treatment. CAP is a partially ionized gas at atmospheric pressure that operates at temperatures below 40 °C^12^, thereby ensuring a gentle treatment that does not cause thermal harm^13^. Plasma generates a complex mixture of reactive oxygen and nitrogen species (RONS), ultraviolet radiation including UV-C, charged particles, and electromagnetic fields, all of which contribute to its diverse properties^14^. CAP has gained increasing attention for its ability to inactivate a broad spectrum of microorganisms, including bacteria^15–17^, fungi^18,19^, and viruses^20,21^, while causing no damage to healthy human cells^1,22,23^. Cold plasma also promotes wound healing by the stimulation of skin cells such as fibroblasts, keratinocytes and melanocytes, activation of biological receptors, and stimulation or regulation of immune cells^24^. It does not pose a risk of allergic or toxic reactions^25^, and can be applied directly to the living tissue without causing pain or damage as reported in various clinical studies^26–28^. In recent years, the application of CAP in medicine has evolved into an innovative field of plasma medicine^29,30^. However, it has mostly been radio frequency (RF) or dielectric barrier discharges (DBD) that were tested for the antimicrobial effects^31–34^. Thus, the potential of microwave plasma sources remains less explored, and further research is needed to evaluate its efficiency and potential applications.

To study the effects of microwave plasma on selected microorganisms, this study examines two plasma discharges: Surfayok (unipolar microwave discharge jet)^35^ and Surfatron (surface‑wave microwave discharge jet)^36^. The study evaluates the inhibitory effects of selected plasma sources based on treatment time, discharge power, enclosure of the treated surface, treatment mode (static or scanning), and, moreover, in view of microbial type: gram‑negative (*Escherichia coli*), gram‑positive bacteria (*Staphylococcus epidermidis*,* Cutibacterium acnes*), and yeast (*Nakaseomyces glabratus*). The model microorganisms were selected as representative pathogens associated with skin infections and chronic wounds^37^. Since, the effects of CAP can be attributed to the synergistic actions of different plasma components. The antibacterial efficacy of CAP is primarily associated with the RONS^38^, charged particles, UV radiation^39^, and the electric field^40^. In this study, four different colorimetric agents (indigo, potassium iodide and Griess assays for nitrite and nitrate ions) were used to investigate the interaction of RONS and UV photons with the treated surface. Biopolymers containing the detection dyes were treated with CAP under different conditions. The results were then correlated with the outcomes of antimicrobial assays performed with *Staphylococcus epidermidis* (*S. epidermidis*), *Cutibacterium acnes* (*C. acnes*), *Escherichia coli* (*E. coli*) and *Nakaseomyces glabratus* (*N. glabratus*). To ensure the reliability and reproducibility of the results, the methodology and analysis were conducted in accordance with E DIN SPEC 91315:2024-08^41^, a novel standard that aims for the unification of plasma research protocols across laboratories. In addition to the “non-direct” testing with biopolymers the effects of CAP were studied using electron microscopy, both scanning (SEM) and transmission (TEM). These methods enabled the study of the morphological and intracellular changes following the CAP treatment.

## Results and discussion

### Static treatment

**Fig. 1:**
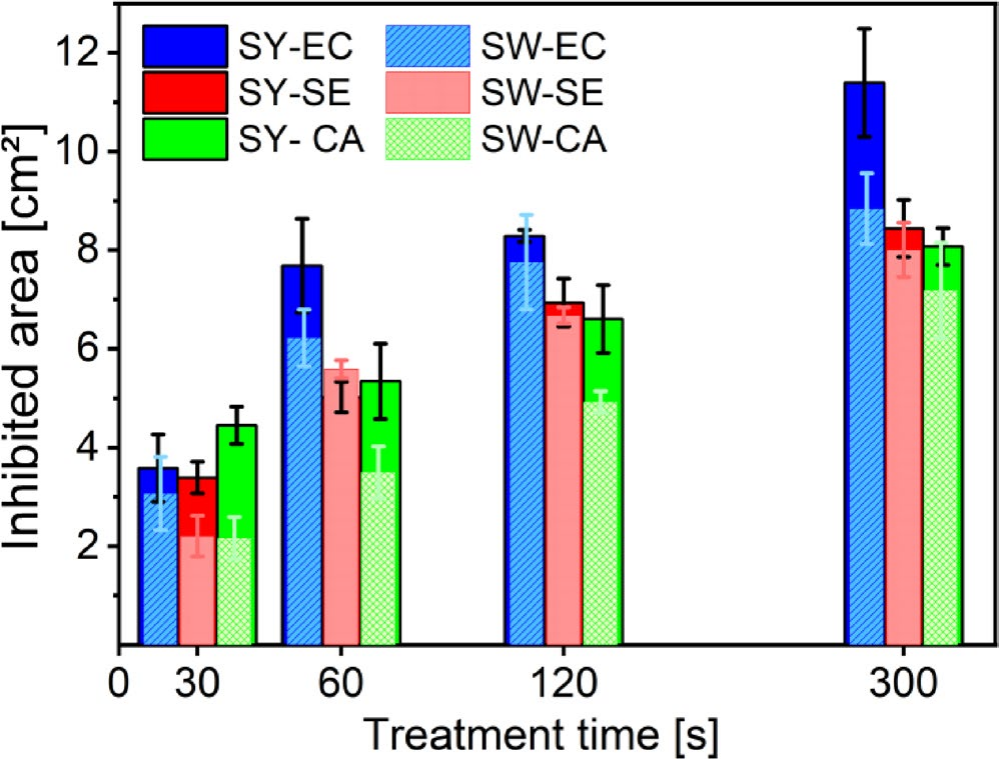
Comparison of inhibition efficacy for: Surfayok (SY - no mesh bars) and Surfatron (SW - mesh bars); against *E. coli* (EC), *S. epidermidis* (SE), and *C. acnes* (CA).

Both MW plasma discharges achieved comparable antimicrobial efficacy in the open-air treatment. The bacterial inhibition occurred from the shortest treatment time of 30 s (Fig. 1). Compared to the untreated or treated with argon gas only controls, where no inhibition was detected. The inhibitory effects increased with increasing treatment time confirming thus the previous experiments done with these devices on the yeast *N. glabratus*^35,42^ as well as the general trend observed in the cold plasma research^31,43^. The continuous increase of treatment efficacy was observed until the treatment time 2 min. Subsequently, the inhibition efficacy increases at a much slower rate due to the limited diffusion of RONS across a broader area in the static treatment mode^19^.

For all tested microorganisms, the inhibited area reached approximately 3–5 cm^2^ after 30–60 s of the treatment. This efficacy is similar for different model microorganisms, highlighting the broad-spectrum efficacy of CAP for decontamination purposes. In comparison with clinically approved plasma jet kINPen^®^ MED, whose recommended protocol suggests a treatment duration of 30–60 s per 1 cm^2^, as stated by the manufacturer^44^. For the MW discharges, the recommendation for the use in the static treatment would be 30–60 s per 3 cm^2^. This represents more than twice the efficacy, which may significantly improve the treatment duration of large chronic wounds. The comparison of the inhibitory effect for all microbial species and different treatment parameters is summarized in the Supplementary Information in Table 3.

#### Open-air vs. enclosed treatment

Based on the previous work with the plasma gun^19^, significantly higher antimicrobial efficacy was achieved for an enclosed treated area. A lid from a Petri dish, with a hole for the plasma outlet was used to simulate an enclosed environment. Thus, the same set-up (see the experimental section) was also tested for the microwave discharges.

**Fig. 2:**
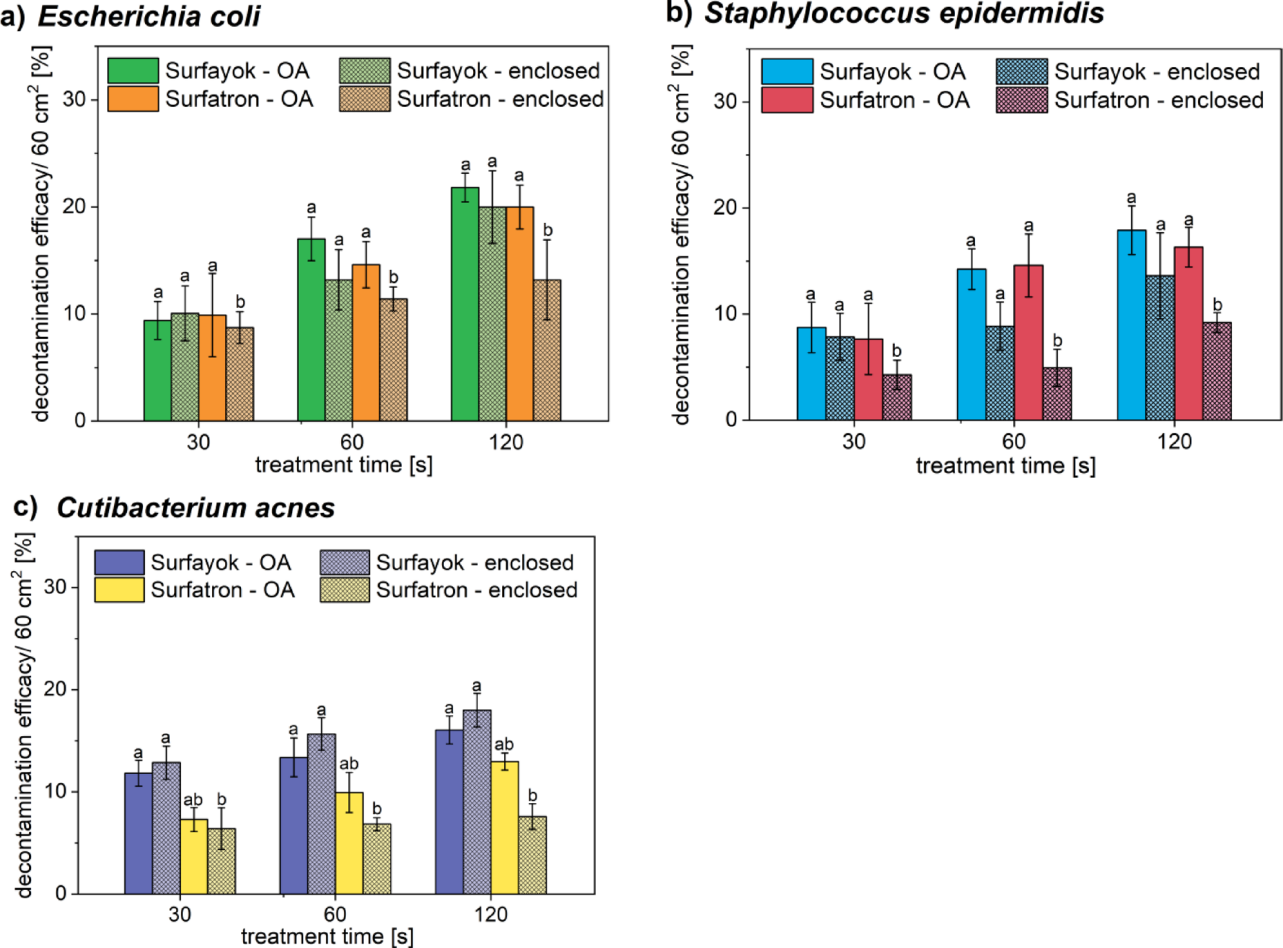
Comparison of decontamination efficacy per Petri dish (60 cm^2^) of enclosed (mesh bars) and open-air (no mesh) treatments against (**a**) *E. coli*, (**b**) *S. epidermidis*, and (**c**) *C. acnes* tested with Surfayok and Surfatron in the static mode.

For the Surfayok (unipolar MW jet), the enclosed treatment had no significant effect on inhibition efficacy across any of the tested microorganisms (Fig. 2). Statistical analyses were performed using STATISTICA (TIBCO Software Inc.) and Microsoft Excel. Data were first checked for normality using the Shapiro–Wilk test and for homogeneity of variances using Levene’s test. Because the data met the assumptions for parametric testing, differences between treatment conditions were evaluated using a two-way analysis of variance (two-way ANOVA) with discharge type (Surfayok vs. Surfatron) and treatment configuration (open-air vs. enclosed) as fixed factors.

Statistical analysis using two-way ANOVA revealed no significant main effect of discharge type on microbial inhibition for *E. coli* ( F (150) = 0.226, *p* = 0.635), *S. epidermidis* ( F (104) = 0.722, *p* = 0.398). Treatment configuration (open-air vs. enclosed) appeared to be more significant, since the p-values were closer to the chosen level of significance α = 0.05. For *E. coli* ( F (150) = 1.179, *p* = 0.279) and *S. epidermidis* ( F (104) = 5.471, *p* = 0.021). For *C. acnes* more statistical significance was found, the effect of discharge type ( F (114) = 2.650, *p* = 0.106), and the effect of the treatment environment ( F (114) = 4.000, *p* = 0.0480).

When significant effects or interactions were detected, Tukey’s honest significant difference (HSD) test was applied for post-hoc pairwise comparisons. Tukey HSD test was confirmed no statistical difference between the studied treatments (open-air vs. enclosed) in case of Surfayok but revealed a significant difference in the treatment configuration for Surfatron, which can be observed from the shortest treatment time (30 s), mainly for more resistant strain of *C. acnes*. All statistical tests were two-tailed, and significance was accepted at *p* < 0.05. Results are reported as mean ± standard deviation unless stated otherwise.

The increased efficacy of the plasma gun^45^ achieved for an enclosed treated area was primarily thanks to the addition of oxygen to the working gas (helium), thus favouring a production of ozone, well-known for its antimicrobial properties. The enclosed treatment prevented oxygen leakage into the surroundings, improving the efficacy of RONS formation and prohibiting their diffusion into the open air. Thus, the RONS could interact more efficiently with the treated surface allowing for better decontamination. In the case of the microwave discharges, neither oxygen nor nitrogen were added to the plasma gas. Pure argon was used as the working gas, which then reacted with the oxygen and nitrogen present in ambient air to form RONS.

In the Surfatron (surface-wave MW jet), the plasma is concentrated into a narrow plasma plume (concentrating the electron density and active particles), and mixing with ambient air occurs primarily at the plume’s edges. By enclosing the discharge under the lid of a Petri dish, the amount of air available for mixing with argon was restrained. Since no fresh air or other reactive gas was introduced into the system, the composition and concentration of RONS, which can interact with the treated surface, remained relatively stable over time. This is mirrored in Fig. 2, which shows that the inhibition efficacy did not increase linearly over time for the enclosed treatment. In contrast when the discharge operates in the open air, argon mixes with the surrounding air, continuously increasing the number of active particles to interact with the treated subject, increasing the inhibitory effect over time.

In the Surfayok (unipolar MW jet), the plasma gas (argon) is blown into the plasma cavity via two inlets. Thus, argon is mixed inside the cavity (producing centrifugal motion, leading to various fluctuations). Thanks to this modulated flow regime, the argon molecules are ionized more efficiently, and plasma is blown out of the cavity perceived as chaotic filaments (see in Materials and Methods). These characteristics ensure efficient mixing of the plasma plume with the surrounding air. Even in enclosed treatment, the discharge can effectively interact with the air leading to a gradual increase in the RONS concentration. The RONS are retained under the lid, allowing for more efficient interaction with the treated surface, similar to the mechanism observed in previous studies using the plasma gun. However, the difference between the treatment with or without the lid for the Surfayok was very small, as no reactive gas was added to the plasma gas. The advantage that comes out from these findings is that the Surfayok can potentially be used to decontaminate also spaces with lower air supply. For example, a treatment of a subject in a confined space (e.g., under a surgical drape).

**Fig. 3:**
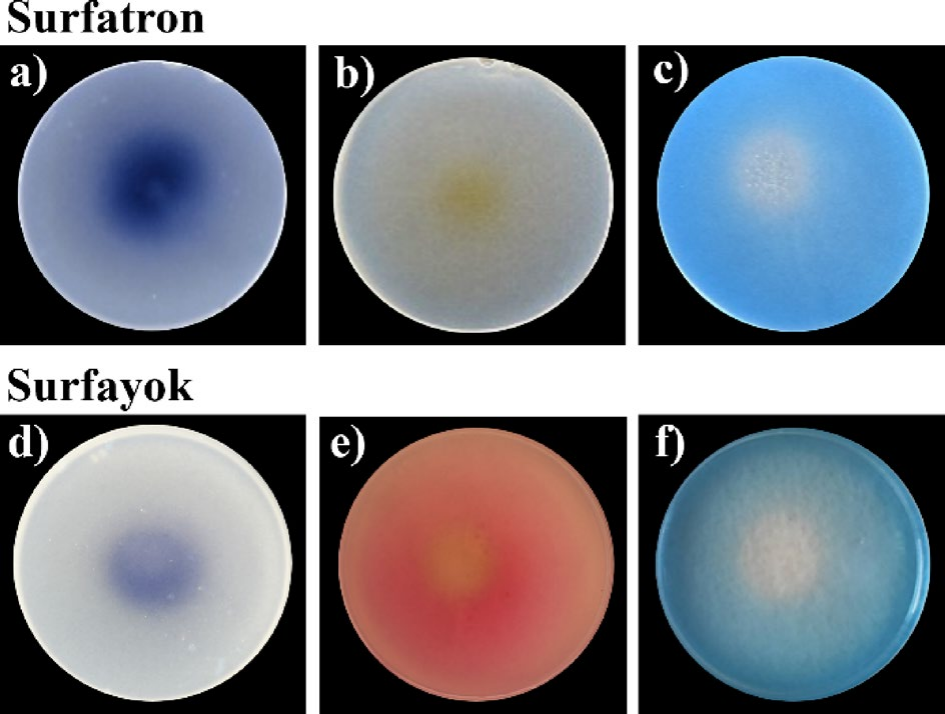
Treatment of biopolymers with colorimetric agents using Surfatron (upper row) and Surfayok (bottom row) for 60 s static treatment a) and d) KI-starch b) nitrate detection e) nitrite detection, c) and f) indigo.

The different antimicrobial efficacy of the Surfatron compared to the Surfayok is thus caused by the Surfayok mixing more efficiently with the surrounding air (producing more RONS), even in enclosed environment. In comparison with the plasma gun^45^, the addition of oxygen admixture to the helium feed gas significantly enhanced generation of RONS and their entrapment under the lid allowed for more effective microbial inactivation in enclosed conditions, where natural mixing with ambient air is limited. Applying a similar strategy to microwave jets may help overcome the limitations observed in narrow discharge geometries like Surfatron when used in confined environments. In contrast to Rf systems, microwave discharges efficiently absorb energy through molecular rotational excitation, which primarily leads to gas heating rather than enhanced RONS production. However, introducing such admixtures into the surrounding environment of the jet may still increase the generation of RONS, particularly under enclosed treatment conditions.

In contrast, the Surfayok discharge produces a more dispersed plasma plume, in which electron density is distributed across multiple transient filaments rather than concentrated within a single channel. This dynamic discharge structure reduces the likelihood of rapid local overheating at the treated surface. Nevertheless, extended static exposure may still result in gradual temperature accumulation. Therefore, scanning or paintbrush-type treatment or intermittent plasma exposure (as previously demonstrated by our group in study Trebulová et al.^42^) represents a more suitable strategy for both effective decontamination and thermal safety when treating living tissues.

#### RONS distribution on the surface

To better understand the differences between the two discharges and their antimicrobial efficacy, it was necessary to characterize the spatial distribution of chemically reactive species on the treated substrate. Different colorimetric agents were therefore incorporated into the biopolymer matrix and served as an essential mechanistic link between the plasma chemistry and antimicrobial efficacy. Unlike UV radiation or thermal effects (which were controlled for and did not produce any detectable inhibition) the biopolymers responded selectively to oxidative reactions, providing indirect but robust evidence that RONS react with the surface in a time-dependent and discharge-specific manner.

##### KI starch – non-selective RO(N)S

A well-known method for the detection of reactive oxygen species is the oxidation of iodide to iodine in a complex with starch. In the KI-starch enriched biopolymers, iodide is non‑specifically oxidised to iodine by the RONS from plasma^46^. The formed iodine reacts with starch to form a complex exhibiting dark blue colour. Thus, the biopolymer´s colour is changing from transparent to dark blue in places where the reaction occurred.

A typical trend of increased stained area in the middle of the biopolymer with increasing treatment time could be observed, corresponding with the microbial assays in the static treatment mode. This can be considered as indirect evidence of the RONS being the major antimicrobial component of the CAP. Using the Surfatron (SW jet), a larger surface area was coloured in shorter time compared to the Surfayok (unipolar MW jet).

The experiments also revealed the “blind spot” of the plasma plume. It was more evident for the biopolymers treated with Surfayok (unipolar MW jet). Fig. 3 shows an unstained spot located directly in the middle of the stained area. This phenomenon likely arises from the limited diffusion of ambient oxygen into the plasma core, where the discharge remains dominated by argon. This may be caused by the modulation in the flow regime mentioned above, causing the gas to form vortex-like pattern creating an eye in the middle, where only a minimum of active particles is present resulting in reduced oxidative activity. In Surfatron, the plasma plume is focused into a narrow beam, with the highest concentration of reactive species located at its tip. As a result, the “blind spot” is significantly reduced, and the most intense colour change appears near the centre, gradually fading toward the edges.

**Fig. 4:**
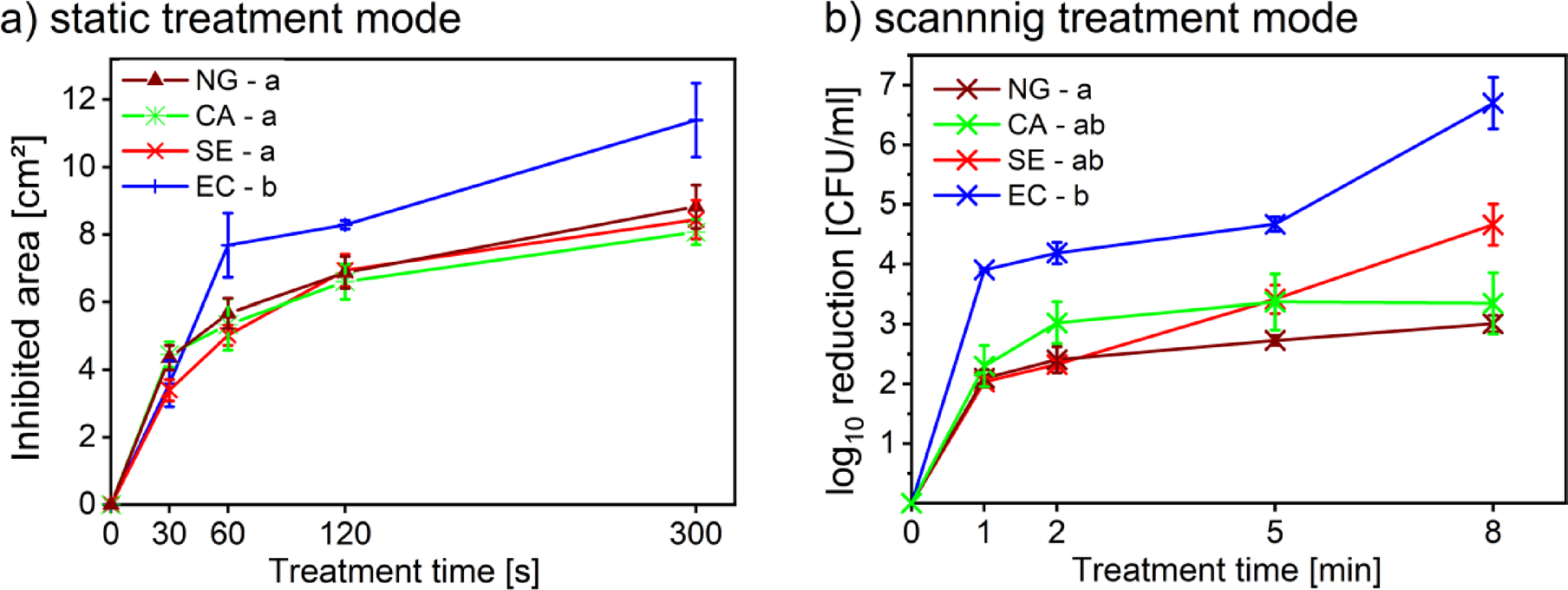
Decontamination efficacy of the Surfayok in static and scanning treatment mode for tested microorganisms: N. glabratus (NG), C. acnes (CA), S. epidermidis (SE) and E. coli (EC).

##### Presence of reactive nitrogen species - nitrates and nitrites – Greiss reagent kit

The presence of nitrates has been confirmed in plasma formed by Surfatron (SW jet), but not in plasma generated by Surfayok (unipolar MW jet). In the Fig. 3b, a yellow-coloured spot is an indicative of the presence of nitrates hitting the surface. It is possible to observe a recurring trend, that with increasing treatment time a larger colour changes occur, as observed in the microbial assays. In case of Surfayok, no evidence of nitrate reacting with the surface was detected for any of the treatment times. This suggests that plasma generated with Surfatron produces a much higher concentration of nitrates compared to the Surfayok plasma.

Conversely, the presence of nitrites (pinkish coloration) was only detected for the plasma generated by Surfayok (Fig. 3e). In the case of Surfatron, no colour change occurred for any treatment time, which may indicate a very low concentration of nitrites in this discharge. In contrast to the highly localized colour changes observed with KI–starch or nitrate indicators, the biopolymers enriched with nitrite indicator dye exhibited a widespread pinkish coloration across a large area with a prominent blind spot in the middle. This trend may be connected to the gas flow modulation and efficacy of mixing with ambient air. Considering the completely opposite results obtained in the RNS test, it can be assumed that in the Surfatron most of the nitrites are oxidised to nitrates, which can then be determined using the nitrate test kit. On the other hand, in Surfayok most of the nitrites remain in this form and therefore the tests for nitrates were not successful.

##### Indigo blue dye - presence of ozone

To improve the selectivity of RONS determination by the dye-enriched biopolymers, indigo blue dye was used. Under optimal reaction conditions, one molecule of indigo is oxidised by approximately one molecule of ozone. Thus, when the indigo dye reacts with ozone, decolourisation, observed as transparency of agar plates^47,48^. However, in case of the plasma treatment, other ROS produced by the plasma may contribute to the oxidization, making the specificity of the reaction more problematic. Nevertheless, the reaction can still be used to determine the presence of ROS (mostly ozone) reacting with the treated surface. The Fig. 3c and f show the discoloured zones in the centre of the agar biopolymers, where the plasma plume was in contact with the treated surface. Using Surfatron, decolourisation of the indigo biopolymer was evident as early as 30 s after the treatment, with a distinct central zone of oxidation clearly visible. In contrast, Surfayok did not show a clearly visible colour change at early time points; however, after prolonged exposure (more than 300 s), the decolourised area became comparable in size to that of Surfatron-treated samples. Notably, Surfayok exhibited greater lateral dissipation of oxidative effects, resulting in a broader, though more gradually developing, treated area. These findings suggest that while Surfatron may induce faster surface oxidation due to its concentrated discharge characteristics, Surfayok enables a more diffused delivery of reactive species over time. The results also align with observations from the open-air versus enclosed treatment setups, where the discharge characteristics influenced the treatment efficacy as discussed in the previous chapter. Unlike the KI–starch biopolymers, which react rapidly and non-selectively to a wide spectrum of oxidative species (and thus, it may magnify early differences), indigo oxidation is more specific and develops slowly, making the spatial and temporal dynamics of each discharge more discernible. This supports the idea that the difference between open-air and enclosed treatments in these systems is influenced not only by RONS production, but also by the dissipation characteristics of each discharge.

Another component of CAP is the UV radiation. To assess the contribution of UV independently from plasma-generated reactive species, control experiments were performed using a UV‑transparent quartz glass barrier placed between the plasma source and the biopolymer surface. A 2 mm thick quartz glass plate was used, which efficiently transmits UV photons while physically blocking RONS, thereby preventing direct plasma–surface interaction. This configuration enabled selective exposure of the biopolymer to UV radiation alone. Using this condition for 10 min the KI and indigo biopolymers remained unchanged, supporting the idea of RONS being primarily responsible for the ongoing reactions causing the colour change in biopolymers.

The observed oxidation patterns mirror the biological outcomes: regions showing strong RONS signatures corresponded to areas of microbial inhibition and help to understand the differences between the studied discharges. The trends observed during the open-air or the enclosed treatment and the assays with dye-enriched biopolymers thus support the idea that RONS are the primary aspect of CAP effects on the target.

After the static spot treatment, scanning of the whole surface area was done to test the distribution of the RONS throughout the whole surface of biopolymer. For both discharges, the blind spots could be eliminated by applying the scanning mode, ensuring homogeneous treatment across the entire surface. The results of these experiments are published in a related study by Loupová et al.^49^.

#### Interaction of the MW discharges with the host tissue

In addition to antimicrobial activity, CAP has been shown to have various beneficial effects on host tissue cells when applied within appropriate therapeutic windows. In eukaryotic cells, CAP-generated RONS act at sublethal concentrations as signalling molecules that modulate redox-sensitive pathways involved in cell migration, proliferation, and wound repair. CAP exposure has been reported to enhance keratinocyte and fibroblast activity, stimulate angiogenesis, and promote re-epithelialization, while excessive oxidative stress is avoided by endogenous antioxidant defences. Moreover, CAP has been shown to influence the local immune response by modulating cytokine release, activating macrophages, and promoting a controlled inflammatory response that supports tissue regeneration ^26,29^. Importantly, these effects are highly dependent on plasma source characteristics, treatment duration, and the spatial distribution of reactive species. Therefore, understanding how different microwave plasma discharges generate and deliver RONS under different conditions is essential not only for antimicrobial efficacy but also for ensuring cytocompatibility and favourable host–tissue responses in future clinical applications.

For instance, in the Surfatron (surface-wave MW jet), the plasma is focused into a narrow plasma plume concentrating the electron density and active particles on the same spot. This configuration enables highly precise, localized application, which can be advantageous for treating small, well-defined areas. However, when the exposure duration exceeds approximately 30 s at 12 W on a single spot, a gradual increase in surface temperature may occur. This temperature rise is partly attributable to the surface-wave excitation mechanism. A standing wave is generated and propagates at the interface of non‑ionized air and plasma. While part of the wave energy is transferred to argon to sustain the plasma, a fraction is dissipated into the surrounding environment as heat. These facts need to be taken into consideration for the potential wound healing treatment, as they could lead to thermal skin irritation. Therefore, for investigation purposes, localised spot treatments over 30 s were used to study the antimicrobial effects and compare the two discharges. However, for medical applications involving treatment of living tissues, a paintbrush‑like movement of the jet over the treated area (10–15 s/cm^2^) is recommended to avoid any thermal damage.

In contrast, the Surfayok discharge produces a more dispersed plasma plume, in which electron density is distributed across multiple transient filaments rather than concentrated within a single channel. This dynamic discharge structure reduces the likelihood of rapid local overheating at the treated surface. Nevertheless, extended static exposure may still result in gradual temperature accumulation. Therefore, scanning or paintbrush-type or intermittent treatment (as previously demonstrated by our group^35^ represents a more suitable strategy for both effective decontamination and thermal safety when treating living tissues. The thermal safety for the host tissue^50^ and positive effects of Surfayok on wound healing^51^ was assessed by Bogdanov et al. in a in-vivo study on mice.

### Scanning treatment

Based on the above findings, the treatment with Surfayok (unipolar MW jet) was chosen as the more potent option for future medical applications, and therefore further measurements were performed with this discharge. Considering the potential applications for wound healing or superficial infection treatment, the scanning treatment was tested to study the treatment time needed for decontamination of a larger area. The scanning parameters and treatment conditions have previously been optimized by Loupová et al.^49^ for the bacterial strains (*E. coli* and *S. epidermidis*), where the results for bacteria are discussed in detail. Thus, this study expands the knowledge by testing the yeast *N. glabratus* and the bacteria *C. acnes* and compares the inhibition efficacy for all the studied microorganisms.

**Fig. 5:**
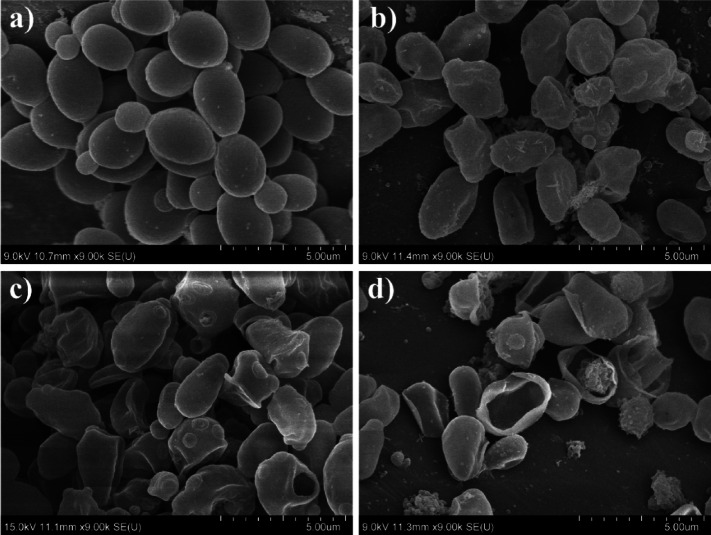
SEM images of plasma treated N. glabratus, for treatment time: a) control b) 30 s; c) 120s; d) 300 s. Note: little spikes in Figure 5b are probably PBS crystals formed during the sample processing.

The samples were prepared in the same manner as for the static treatment. However, instead of maintaining the plasma plume at a fixed point, it was continuously moving across the entire agar surface. After 24 h of the post-treatment cultivation gradual reduction in colony counts with the increasing treatment duration was observed, consistent to the results of the static mode (Fig. 5). In agreement with other studies in the field^31^, higher inhibitory effects were achieved for gram‑negative bacteria *E. coli* compared to the gram-positive *S. epidermidis* and *C. acnes* and the yeast *N. glabratus*. The results of static plasma treatment on different microorganisms, shows that *E. coli* exhibited the highest sensitivity to CAP, showing the largest increase in inhibited area with longer treatment time (Tukey’s HSD test). For the remaining model microorganisms (*N. glabratus*, *C. acnes* and *S. epidermidis*), the values of inhibited areas were very similar. Although *E. coli* showed the largest inhibited area across time, the difference was not statistically significant compared to other species (ANOVA, F (3,63) = 1.346, *p* = 0.267). A similar trend observed for the static mode can be seen for the scanning, where also only *E. coli* showed higher susceptibility to the plasma treatment (Tukey’s HSD test), while the other microorganisms were more resistant. Notably, *Escherichia coli* exhibited a pronounced increase in inactivation after 8 min of scanning treatment. We hypothesize that this effect may be related to the cumulative plasma dose delivered during continuous scanning of the entire agar surface. Thus, the sharp increase in inactivation efficacy may reflect a dose‑dependent transition from sublethal oxidative stress to irreversible membrane and cell wall damage. The results obtained for the scanning mode analysed via statistical apparatus revealed a significant difference between the susceptibility of different microbial species (ANOVA, F (3,171) = 4.808, *p* = 0.020). Thus, for both treatment modes, these findings support the hypothesis that gram-positive bacteria are generally more resistant to CAP than gram‑negative bacteria; further they indicate, that yeasts exhibit overall greater resistance compared to bacteria.

The results of scanning treatment obtained for both *C. acnes* and *N. glabratus* show that the lowest number of colonies was recorded following the longest exposure time of 8 min. However, when considering all the treatment times, the most substantial decrease in colony numbers occurs just after 2 min of the treatment. However, when comparing the results of this study with the references study by Loupová et al. ^49^ *N. glabratus* and *C. acnes* appeared more resistant compared to *E. coli* and *S. epidermidis*. Even after 8 min of the CAP exposure, complete decontamination was not achieved. This reduced efficacy may be partly attributed to the higher initial inoculation concentration used for *C. acnes* and *N. glabratus* compared to the bacteria tested in the referenced study by Loupová et al. ^49^. For *N. glabratus* and *C. acnes*, higher initial inoculation concentrations were deliberately employed to assess potential multilayer formation and its impact on plasma treatment efficacy, whereas bacterial data were included from a previously published dataset optimized for different experimental objectives. The higher initial inoculation concentration may have resulted in the cells forming multiple layers rather than a uniform monolayer. This layered arrangement of yeast cells adhering to the agar surface is likely the cause of the observed decrease in inhibition efficacy after 2 min of the treatment in scanning mode. The uppermost cell layer is directly exposed to plasma and therefore, most affected. The damaged or necrotic cells may form a physical shield over underlying layers, protecting them from the plasma exposure. This effect likely explains why the expected linear reduction in viable cells did not continue beyond two minutes of the treatment. Thus the 2‑minute treatment offers an optimal balance between efficacy and treatment duration for given area. These findings are further supported by SEM observations discussed later in the paper.

Nevertheless, the results confirm the broad-spectrum antimicrobial efficacy of the CAP treatment in both static and scanning modes. Depending on the size and nature of the infected area, the appropriate mode can be selected, and the treatment time can be adjusted to achieve optimal decontamination efficiency.

#### SEM and TEM

To investigate the structural effects of the plasma treatment, selected samples were analysed using scanning electron microscopy (SEM) and transmission electron microscopy (TEM). The Surfayok device operated in the scanning mode was chosen for these experiments, as the previous results demonstrated its highest inhibition efficacy among the tested configurations. *N. glabratus* was selected as the model microorganism due to its relatively higher resistance to the plasma treatment, compared to the studied bacteria. It has also been chosen because several studies have reported SEM observations of plasma treated bacteria, but only a few have examined yeast species such as *Saccharomyces cerevisiae*. Therefore, this work provides valuable new insight into the morphological response of *N. glabratus* to the CAP treatment.

##### Morphological changes

**Fig. 6:**
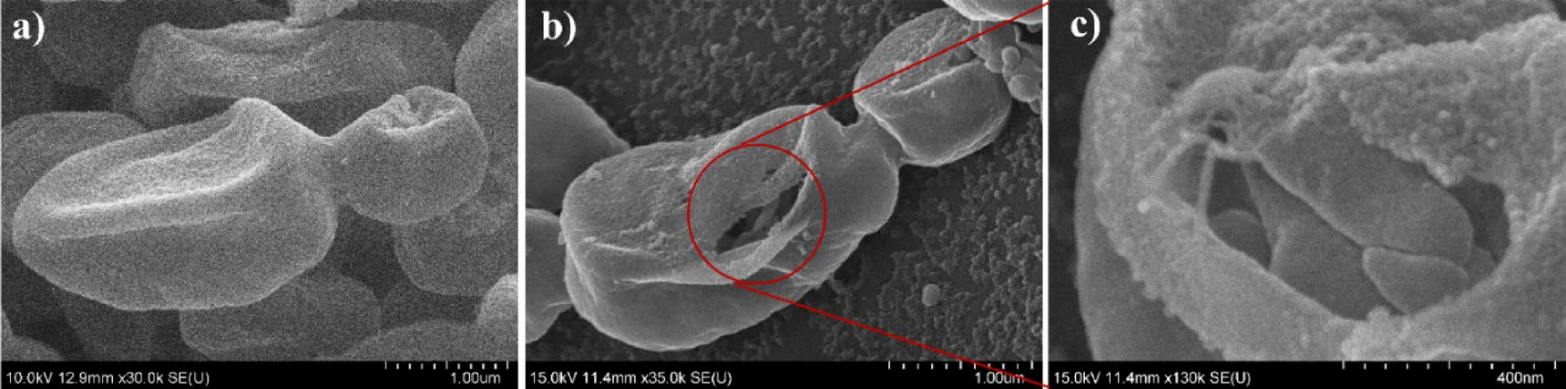
Cells of N. glabratus after (a) 60 s (b) and (c) 120 s CAP treatment.

The SEM images of the plasma treated *N. glabratus* revealed progressive morphological changes in microbial cells correlated with the increasing treatment duration (Fig. 6).

After 30 s of CAP exposure, cells showed initial signs of stress, including shrinkage and surface texture changes. While some cells retained their oval shape, others exhibited surface protrusions and signs of morphological collapse (Fig. 5b).

At 60 s (Fig. 5c and a), more pronounced damage was observed, particularly a visible thinning of the cell wall, likely resulting from the ion bombardment or “etching” action of RONS, which may gradually degrade the outer cellular layers. *N. glabratus* possesses a structurally rigid cell wall composed of chitin, β-glucans, and mannoproteins^52^. Our observations of localized structural disruptions may indicate oxidative modification of mannoproteins or β-glucans, localized etching or polysaccharide rearrangement. Similar early‑stage morphological disruptions have been reported in plasma-treated bacteria. In bacteria, CAP-induced morphological changes, such as surface roughening, blebbing, and the appearance of nano-scale protrusions often attributed to oxidative damage of membrane lipids and proteins^53^. Dezest et al.^54^ observed pronounced membrane deformation and leakage in *E. coli*, including cell rounding and loss of rod morphology following the CAP exposure, likely due to the combined effects of RONS and electric field. Barkhade et al.^55^ further demonstrated severe surface alterations in *Staphylococcus aureus* and *Salmonella abony* after microwave plasma treatment, including membrane thinning, blebbing, and rupture, correlated with elevated levels of hydroxyl radicals and hydrogen peroxide. Although yeast cells possess a thicker and more rigid cell wall, these results suggest that the CAP treatment may induce nano-scale surface reorganization even in more robust cell wall of fungi.

By 120 s, structural variability among cells increased. While a few cells remained largely intact, the majority displayed deformation in both shape and surface texture, consistent with earlier observations. Notably, approximately 40% of cells showed clear signs of membrane rupture, with cytoplasmic leakage evident (Fig. 6b and c).

**Fig. 7:**
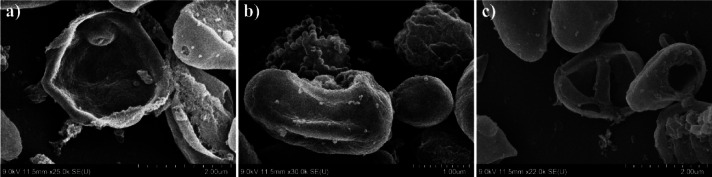
Cells of N. glabratus after 300 s CAP treatment.

At 300 s, extensive damage was apparent. Most cells appeared non-viable, with their structural integrity severely compromised. Many were reduced to collapsed cell wall remnants (ghost cells) surrounded by leaked intracellular content (Fig. 7a and b). While most cells exhibited a single hole in the cell wall, few cells showed multiple perforations (see Fig. 7c).

**Fig. 8:**
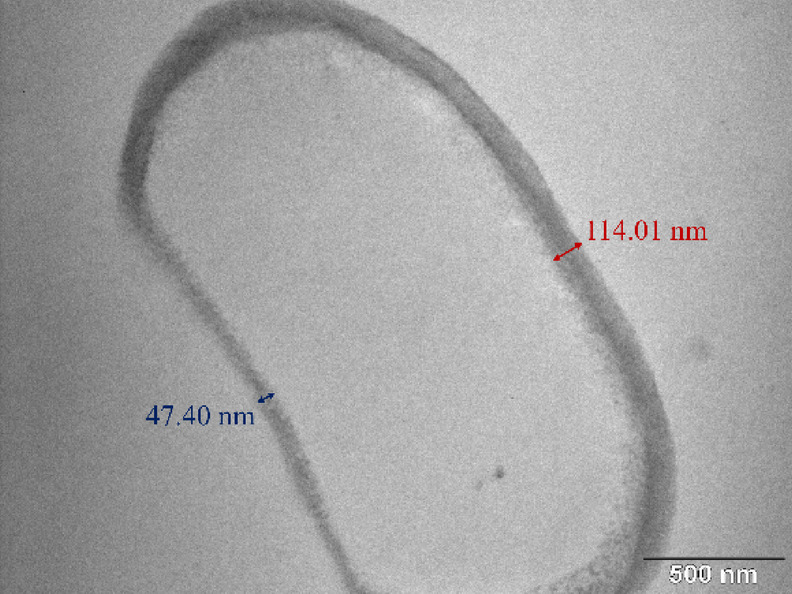
One-sided thinning of the cell wall after 5 min CAP treatment visualized by TEM.

Given that yeast cells were treated while adhered to a Petri dish surface, it is plausible that the side of the cell facing the plasma jet received higher RONS flux and electromagnetic exposure. This immobilized exposure could result in asymmetric stress, consistent with the observation that damage frequently occurred on the side of the cells. Thus, localized thinning of the cell wall, as evidenced by both SEM surface deformation and TEM cross-sectional imaging can be seen (see Fig. 8). This effect may also be linked to the results of the viability reduction after the scanning treatment mode, where the “shielding” action of necrotic cells was reflected in the plateauing of inhibition efficacy after two minutes of the CAP exposure in scanning treatment. SEM images revealed numerous ghost cells and several apparently intact cells, suggesting that viable cells may have been physically protected beneath the overlying layer of damaged cells. In such cases, RONS could not effectively penetrate to the lower layers, reducing the overall treatment efficacy.

**Fig. 9:**
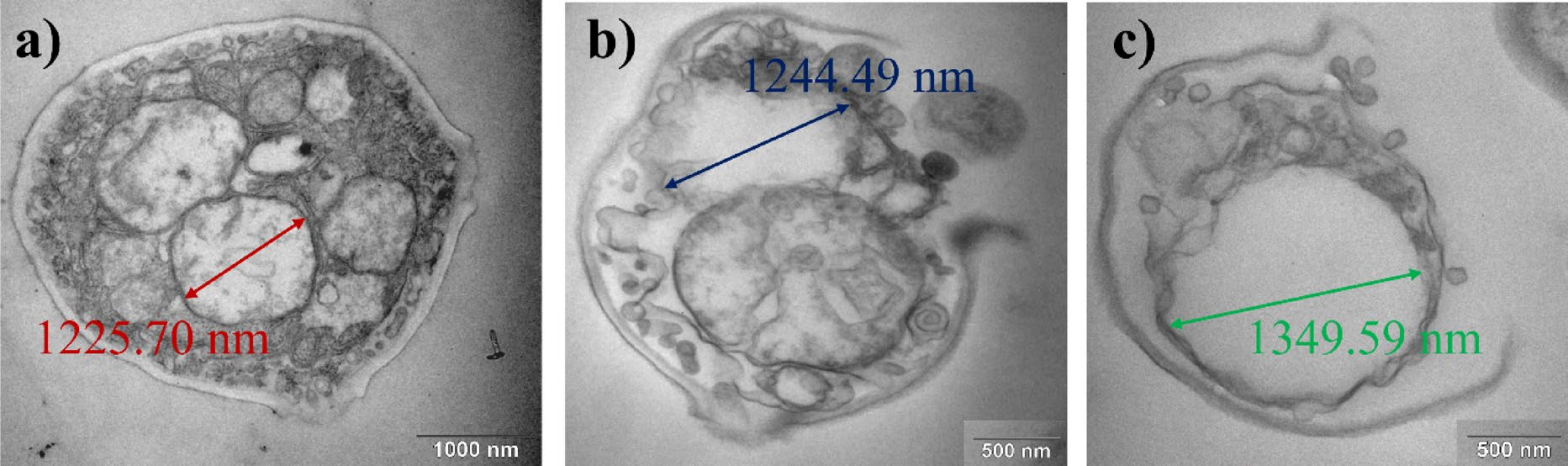
Changes of intracellular content arrangement and structure a) control b) 1 min CAP, c) 5 min CAP treatment.

The shielding effect observed in multilayered microbial populations is highly relevant for clinically realistic wound environments, where microorganisms frequently exist within structured biofilms or layered tissue-associated communities. In such systems, RONS predominantly affect the most superficial layers, while underlying cells may be partially protected by necrotic debris, extracellular polymeric substances, or damaged cell envelopes. This shielding can limit the effective penetration depth of short-lived species and lead to a plateau in antimicrobial efficacy during prolonged treatment. Intermittent CAP treatment combined with intermediate washing steps may represent a promising approach to remove inactivated surface layers and improve the delivery of RONS to deeper microbial layers.

After 480 s treatment the majority of cells appeared as ghost cells, characterized by empty cell walls lacking intracellular content, often surrounded by aggregated protoplasts or leaked cytoplasmic material. Only a small fraction of intact cells remained, and among them a few displayed swelling features occurred. These swollen cells were notably larger in size (approx. 5 μm) and exhibited a uniformly smooth cell wall surface. Such swelling may hypothetically reflect a stress response, possibly linked to osmotic imbalance or water influx caused by plasma‑induced membrane weakening. However, in the absence of further experimental evidence, this interpretation remains speculative.

##### Intracellular changes

With increased treatment time, a clear progression of the cell damage can be observed - from the surface etching to the cell wall perforation, and ultimately cytoplasmic membrane rupture accompanied by leakage of intracellular content. It may seem from the SEM images, that only the longer treatment times cause severe damage to the yeast cells, however, the TEM images have provided a deeper insight into the problematics.

Since CAP triggers an oxidative stress response, one of the earliest and most conserved responses to oxidative stress is vacuolar remodelling. Under oxidative or nitrosative stress, yeast vacuoles expand in size due to the fusion of smaller vesicles, increased autophagic flux, and accumulation of oxidized metabolites destined for degradation^56^. The observed vacuolar enlargement in *N. glabratus* after CAP exposure is thus consistent with an activated autophagic‑vacuolar detoxification pathway, where damaged organelles and oxidized proteins are sequestered for recycling (Fig. 9).

**Fig. 10:**
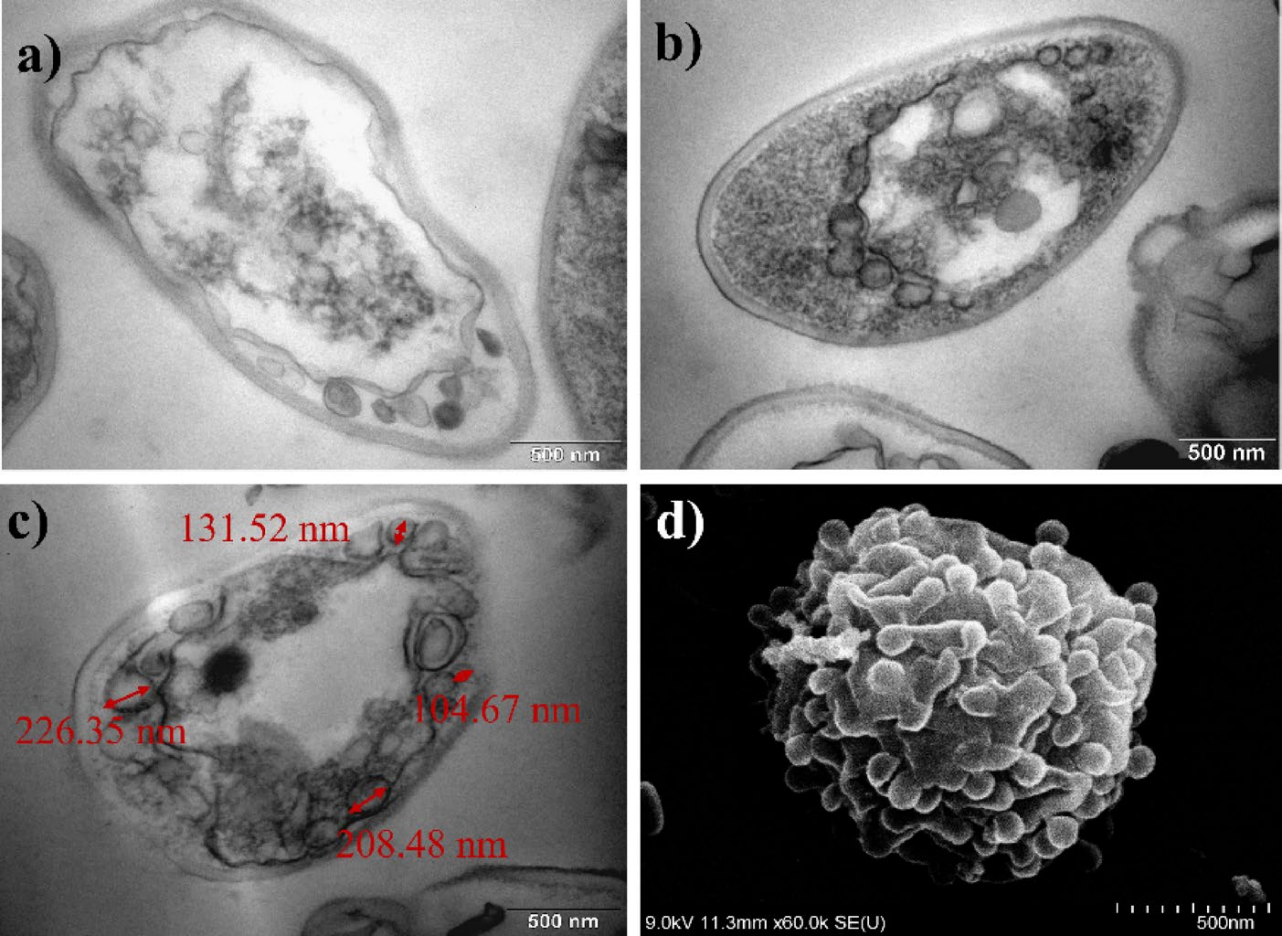
Cytoplasmic membrane shrinkage in N. glabratus a) control b) after 1 min CAP treatment c) release of small vesicles into the periplasm after 1 min CAP treatment d) aggregated protoplasts of N. glabratus after 2 min CA treatment.

After short treatment times, cells exhibited morphological alterations at the cell wall, such as localized protrusions, irregular surface features, and minor shrinking. These changes, however, were relatively subtle compared to the profound modifications observed in the intracellular space. TEM cross-sectional imaging revealed that, despite the robust outer morphology, the cytoplasmic membrane had retracted considerably, with the cytoplasmic volume reduced to nearly half of that observed in untreated cells (see Fig. 10a and b).

**Fig. 11:**
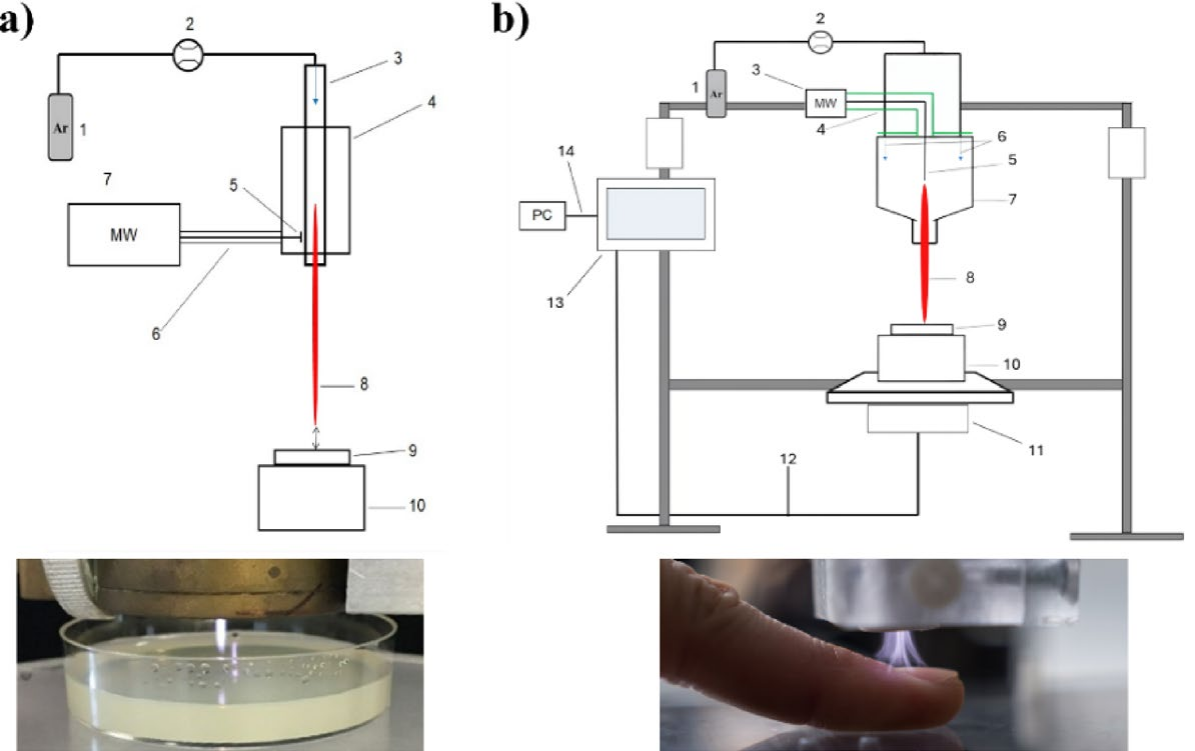
Experimental set-up ​35​ a)surface-wave MW jet SURFATRON; b) unipolar MW jet SURFAYOK; 1 - argon source; 2 - mass flow controller; 3 - MW source; 4 - MW coaxial cable; 5 - free ending of the coaxial cable acting like antenna; 6 - direct argon supply; 7 - resonance cavity, 8 - plasma plume; 9 - Petri dish; 10 - Petri dish holder, 11 - movable plate; 12 - connection of the movable plate with computer; 13 - motor for the movement of the movable plate (11) and plasma device, 14 - computer.

In response to the CAP treatment, we have also observed the creation of small vesicles (Fig. 10c). The small vesicles (100–250 nm) were visible on the outside of the plasma membrane, suggestive of extracellular vesicle release or extrusion of intracellular content. In the yeast cells, peroxisomes are the key ROS‑detoxifying organelles; yet, under the CAP stress they may rapidly become overloaded. We hypothesize that this could signify an oxidative stress induces pexophagy, a selective form of autophagy targeting peroxisomes. Thus, rather than expelling intact peroxisomes, the cell responds by budding off peroxisome-derived vesicles (PDVs) enriched in antioxidant enzymes such as catalase and peroxidases. In yeasts, the Atg proteins (Atg1 (CgAtg1) for *N. glabratus*) mediate the recognition of overloaded peroxisomes and their delivery to the vacuole for degradation^57^. However, when the vacuolar system is overloaded (as could occur under strong plasma-induced oxidative stress), cells may divert part of this autophagic cargo toward secretory autophagy, releasing small vesicles into the periplasmic or extracellular space^58^. These vesicles may contain antioxidant enzymes, such as catalase or peroxidases, and serve as a defensive adaptation to neutralize extracellular RONS before they can penetrate further into the cytosol. Besides peroxisome-derived vesicles involved in oxidative detoxification, yeasts are known to produce other extracellular vesicles (EVs) as part of their stress and communication systems^59^. In *Candida albicans* and *Saccharomyces cerevisiae*, oxidative and osmotic stress were shown to stimulate the formation of stress-induced vesicles carrying antioxidant enzymes (such as superoxide dismutase and catalase), heat shock proteins, and signalling molecules ^60,61^. These vesicles are thought to act both as protective factors, scavenging extracellular ROS, and as quorum signalling agents, allowing neighbouring cells to sense and prepare for oxidative conditions. In the context of CAP treatment, where high local RONS and electromagnetic fields disturb membrane potential, such vesicle release may therefore serve a dual function: first, to export oxidized or excess cellular material (preventing intracellular RONS accumulation), and second, to signal to adjacent cells within microbial populations about the oxidative threat. The vesicular structures observed in TEM thus may represent a heterogeneous population (peroxisome‑derived vesicles (PDVs), autophagic vesicles, and extracellular signalling vesicles) collectively reflecting a multifaceted plasma stress response.

The vesicles appeared to subsequently aggregate on the plasma membrane, contributing to the formation of larger condensed protoplast structures surrounded by attached vesicular debris. The intracellular content frequently appeared in both SEM and TEM as small, rounded structures (< 1 μm) shown in Fig. 10d, either fully expelled from or still partially enclosed by the cell wall. These photos may signify that under the influence of CAP, the intracellular content loses its native organization and condenses into tightly packed aggregates. In several cases, the round structures remain visually connected to ruptured cell wall openings, resembling a partial extrusion or expulsion of cytoplasmic material. This morphology supports the hypothesis that the inner cellular components undergo oxidative crosslinking, osmotic collapse, and structural reorganization following membrane rupture. Thus, we may assume that internally structured spheres likely represent compacted protoplast remnants. Their tightly arranged appearance suggests that oxidative stress and water loss lead to the aggregation of denatured proteins, ribosomal debris, and cytoskeletal components into a thermodynamically favourable spherical shape. Similar plasma-induced protoplast aggregation and cytoplasmic collapse have been observed in *Saccharomyces cerevisiae* by Ravash et al.^62^, further supporting this interpretation.

In addition to already reported to morphological changes and intracellular damage, alterations to genetic material need to be taken into consideration. CAP can induce lipid peroxidation, producing relatively long-lived secondary products such as malondialdehyde, which are capable of diffusing into the cell interior and reacting with proteins and nucleic acids. Such secondary oxidative messengers have been shown to amplify intracellular damage, including protein modification, DNA strand breaks, and DNA – protein crosslinks^63^. Previous studies in eukaryotic microorganisms such as *Saccharomyces cerevisiae*^64^ have demonstrated that CAP exposure may lead to DNA lesions, including single and double-strand breaks, as well as DNA – protein crosslinks, which activate DNA damage response and repair pathways. Similarly, genome-wide analyses performed on *Candida albicans*^65^ surviving sublethal CAP exposure revealed the presence of single nucleotide variants, insertions, and deletions. However, it should be noted that genetic mutations are a natural response of cells to any stress^66^ and the changes caused by CAP were not associated with increased virulence or drug resistance.

While the present study did not directly assess plasma-induced genotoxicity, such intracellular damage may contribute to the observed antimicrobial effects, particularly at longer treatment times or higher plasma doses. Importantly, existing literature suggests that although CAP can induce DNA damage in surviving cells, these changes are generally limited and do not necessarily translate into adverse phenotypic consequences. Future studies combining biochemical assays and microscopic analysis will be required to study the relative contribution of genetic damage to microbial inactivation under the plasma conditions employed here.

## Discussion and conclusions

This study demonstrates the antimicrobial efficacy of cold atmospheric pressure plasma (CAP) generated by microwave (MW) plasma jets, highlighting its potential application in medical decontamination, particularly for wound treatment. Various plasma treatment modes were tested to establish the optimal treatment conditions. Both plasma discharges achieved comparable antimicrobial efficacy, demonstrating their potential as non-chemical alternatives for infection control. Thus, the study extends knowledge on the microwave plasma sources from their high-power use (e.g., wastewater treatment) to potential low-power medical applications. The inclusion of MW discharges into medical field may thus complement the performance of currently available plasma systems, such as kINPen^®^ MED^44^, PlasmaDerm^67^, PlasmAction^68^ and others which are already in clinical use by reducing the required treatment time, potentially improving the economic feasibility of CAP therapy.

Both the static and scanning treatment modes showed a time-dependent increase in antimicrobial efficacy, consistent with findings from previous CAP studies^19,35,43^. However, in the static mode, the inhibitory effect plateaued after approximately two minutes of the exposure. This was likely due to the limited diffusion of RONS away from the localized treatment site, reducing the benefit of longer exposure times. In contrast, the scanning mode continuously redistributes the plasma plume across the surface, allowing more uniform RONS delivery and making it particularly advantageous for treating larger surface areas. Based on these observations, the static treatment may be well-suited for localized or confined infections, while the scanning mode offers a more efficient approach for the decontamination of larger wounds or irregularly shaped surfaces. However, even in the scanning mode, considerable decline in the treatment efficacy was observed after 2 min of the treatment. This reduced efficacy was likely influenced by the layered arrangement of microbial cells on the agar surface, where necrotic upper layers shielded underlying cells from further damage. In the clinical scenario, one could avoid this problem by washing the wound after each minute of scanning to remove the necrotic cells and to prevent the shielding effect.

Among the tested operational modes, the Surfayok jet in the scanning mode exhibited the highest inhibition efficacy across all tested microorganisms, including *Staphylococcus epidermidis*, *Escherichia coli*, *Cutibacterium acnes*, and *Nakaseomyces glabratus*. In a control experiment where only the carrier gas was applied without igniting the plasma, no inhibition was observed, confirming that the gas itself is non-toxic and that the antimicrobial effects arise specifically from the plasma-generated components.

When comparing open-air and enclosed treatment configurations, the Surfayok jet maintained high antimicrobial efficacy regardless of enclosure, likely due to its broader plasma plume and efficient mixing with ambient air. However, results from the Surfatron jet demonstrated that enclosing a narrow, focused plasma jet can significantly limit air-plasma interaction, leading to reduced RONS generation and to a stagnation of antimicrobial efficacy over time. It underscores the importance of allowing sufficient gas-phase mixing, especially when applying CAP (supplied by noble gas, only) in semi-enclosed or fully enclosed clinical environments, such as under surgical drapes or inside wound dressings These findings highlight the potential benefits of supplying small amounts of reactive gases (such as oxygen or nitrogen) into the vicinity of the plasma plume, rather than directly into the microwave discharge itself.

To better understand the plasma‑surface interactions and spatial distribution of RONS, agar‑based biopolymers enriched with colorimetric agents were employed. These visual indicators confirmed that RONS are predominantly responsible for microbial inactivation, with effects increasing over exposure time and correlating with the treatment configuration. The differences between the two studied discharges were underlined using the indigo dye, where higher dissipation of ozone was observed for Surfayok and in the production of RNS, where nitrites were only detected for Surfayok and conversely nitrites only for Surfatron. Experiments performed with the indicator dyes also demonstrated that UV radiation in the absence of plasma-generated reactive species did not lead to the chemical reaction with the dye, neither to significant microbial inhibition. In these control experiments, the treated surface was separated from the plasma plume by a UV-transparent silica glass barrier, which allows transmission of UV photons while effectively blocking charged particles and reactive plasma species. These results reinforce the conclusion that UV radiation does not play a primary role in the antimicrobial efficacy of CAP under the investigated conditions. Reinforcing the conclusion that UV radiation does not play a primary role in the antimicrobial efficacy of CAP.

Finally, scanning and transmission electron microscopy (SEM and TEM) revealed the progressive structural damage in *N. glabratus* cells after the plasma treatment. The morphological changes include cell surface protrusions and signs of morphological collapse, localized cell wall thinning, membrane rupture and pore formation. Similar oxidative modifications of the cell envelope have previously been reported in CAP‑treated bacteria^54,55^. Another notable observation was the emergence protoplast aggregation (dense, spherical aggregates (< 1 μm)) either fully expelled or still partially enclosed by the cell wall. These structures likely formed through oxidative crosslinking, osmotic collapse, and cytoplasmic reorganization after membrane rupture; consistent with earlier reports in *Saccharomyces cerevisiae*^62^. An enlargement of vacuole size with the increased treatment time and formation of vesicle like-bodies were also apparent. Such vesicles peroxisome‑derived vesicles (PDVs) enriched in antioxidant enzymes as well as signalling vesicles and perhaps others, may act as a temporary “ROS buffer” in the periplasmic space but may also serve as quorum sensing agents. The vacuolar enlargement and increased appearance of vesicles in the CAP‑treated *N. glabratus* likely represent the combined activation of oxidative stress induced vacuolar expansion, autophagic vesicle release and secretory autophagy. These findings offer further insight into the downstream effects of RONS not only at the membrane level, but also within the cytoplasmic compartment of affected cells. Based on the SEM and TEM imaging, we hypothesize that the progressive structural degradation of *N. glabratus* is driven by the synergistic action of RONS and electromagnetic fields (including microwaves) during CAP exposure. Altogether resulting in cell wall thinning, pore formation and membrane rupture, vacuole enlargement, vesicle formation, protoplast aggregation and cytoplasmic leakage.

To conclude, the results of this study not only support the effectiveness of microwave plasma jets as a promising non‑thermal decontamination strategy but also provide valuable mechanistic insights into plasma‑microbe interactions, paving the way for further exploration of CAP in clinical settings. Future work will focus on evaluating cytocompatibility and biological safety of the investigated plasma sources, including systematic testing on eukaryotic human cell lines and in vivo testing. Such studies will be essential for assessing the translational potential of microwave-driven CAP systems for biomedical applications.

## Materials and methods

### Preparation of agar biopolymers enriched with colorimetric agents

For the preparation of biopolymers, 2.5 g of agar was added to 100 ml of distilled water. The mixture was heated to 85 °C and stirred continuously for 5 min. Afterwards, the heater was turned off, and the mixture was left to cool down to 60 °C. Once the temperature was reached, appropriate colorimetric agents was added. The following reagents were embedded into the biopolymer structure to determine the studied reactive species by their colour change: indigo dye (0.001 g) for ozone, potassium iodide (0.5 g) with starch (0.4 g) for non-specific ROS, and Merck´s Griess reagent powder kits (according to the manufacturer´s guidelines) for nitrates and nitrites. The amounts are listed for the volume of 100 ml of distilled water with 2.5 g of agar. Once the indicator dye was homogenized with the agar base, this mixture was poured into Petri dishes (*Ø* = 55 mm). Solidified agar plates and were used for the CAP treatment.

### Preparation of microbial samples

To observe the decontamination effects several bacteria namely *Escherichia coli* CCM 3954, *Staphylococcus epidermidis* CCM 4418, *Cutibacterium acnes* CCM 3344 and a yeast *Nakaseomyces glabratus* CCM 8270 were chosen. All the microbial species were supplied by the Czech Collection of Microorganisms, Masaryk University Brno. Inocula for all microorganisms were prepared in 25 ml of a sterile, liquid medium. Brain Heart Infusion (BHI) Broth (Himedia) was prepared for *S. epidermidis* and *C. acnes*, Lennox Broth (LB) (Sigma‑Aldrich) for *E. coli* and YPD (Yeast extract - Peptone - Dextrose/Glucose) medium for *N. glabratus*. The inocula were incubated in a shaking thermostat at 37 °C for 18 h to obtain microbial cultures for the testing of plasma treatment.

To test the static treatment mode, the prepared inoculum of studied microorganism was diluted with a sterile PBS buffer to a desired concentration (10^6^ CFU/ml). The turbidity of the diluted inoculum was measured using a NanoPhotometer‑Implen spectrophotometer at the wavelength of 630 nm. The concentration was calculated according to the calibration equation (see Table 2 in the Appendix), measured for each microorganism specifically. The diluted microbial culture (10^6^ CFU/ml) was inoculated (100 µl) onto the agar plates with the diameter of 90 mm. Immediately after inoculation, the samples were treated with a selected discharge (Surfayok or Surfatron). To test the scanning treatment mode with *N. glabratus*, 50 µl of the diluted inoculum (approx. 10^5^ and 10^6^ CFU/ml) was inoculated onto the agar plates with the diameter of 55 mm. Immediately after inoculation, the samples were treated with the selected discharge (Surfayok).

### Plasma treatment

Both microwave plasma systems Surfatron (Fig. 11a) and Surfayok (Fig. 11b) generated plasma at atmospheric pressure with power of 12 W and the argon flow rate of 5 Slm. Detailed description of the tested plasma sources and optimalization of the treatment conditions can be found in the previously published study^35^. Prepared biopolymer plates, containing reagents for RONS determination or microbial culture for decontamination efficacy testing, were placed under the plasma jet. The agar plates were placed perpendicular to the plasma discharge, so that the tip of the plasma plume was touching the biopolymer surface in the centre, ensuring the highest concentrations of RONS spreading on the treated surface.

The samples were treated by the cold plasma torch at different treatment times and modes (static or scanning). The power of 12 W was used, and treatment times were 30 s, 60 s, 120 s, 300 s (and 480 s for scanning). Based on previous measurements^19^ two different settings of the plasma set-up were tested. Firstly, an open-air treatment and secondly, an enclose treatment, where a lid from a Petri dish with a hole inside (the size of the plasma tube outlet) was used to cover a series of the treated samples. Inoculated, non-treated Petri dishes as well as Petri dishes treated with gas flow only, were left as control samples for each experiment. All measurements were performed in 3 biological replicates composed of 3 technical replicates for each treatment condition. The treatment conditions are summarized in Table 2.

**Fig. 12:**
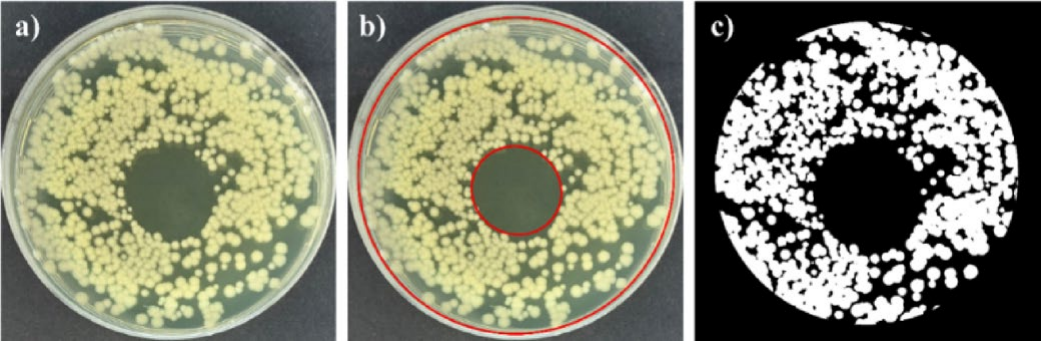
Evaluation of inhibition area size via Power Point (**b**) and software Aurora (**c**) for C. acnes treated for 60 s with Surfayok.

After the treatment, the samples were cultured at 37 °C for 24 h and photographed. To calculate the inhibited area in cm^2^, the Microsoft Power Point was used. For each photo an ellipse or a circle (the dimensions of which were recorded) was drawn across the inhibited area and the corresponding Petri dish. From the recorded dimensions, the areas of ellipses corresponding to the inhibited areas and Petri dishes were calculated. The calculated areas were subsequently converted to the actual dimensions of the Petri dishes with the diameter of 5.5 cm (see Fig. 12b). To determine the antimicrobial efficiency and to calculate the number of survival colonies, the photographs were evaluated in collaboration with HexTech Research s.r.o. using the software Aurora^69^(see Fig. 12c).

Results were also analysed using the Statistica software to evaluate the significant differences among the studied conditions. According to the data characteristics, appropriate tests were chosen to calculate the value of significance (p-value) with the significance level *α* = 0.05.

**Table 1:** Treatment conditions.

Testing condition	Surfatron	Surfayok
Power [W]	12	
Working gas	Argon 4.6	
Gas flow rate [Slm]	5	
Treatment time [s]	30, 60, 120, 300	
Treatment distance [mm]	tip of the plasma plume touching the surface (approx. 1 cm from the nozzle edge)	
Treatment mode	Open air treatment	
	Enclosed (lid) treatment	

### Preparation of samples for SEM and TEM

To investigate the morphological changes in microbial cells following plasma exposure, the Surfayok plasma jet was used in the scanning treatment mode with *N. glabratus* as the model organism. Before the plasma treatment, 50 µl of an undiluted *N. glabratus* culture (~ 10^7^ CFU/ml) was inoculated onto 55 mm diameter agar plates. Then, the samples were immediately exposed to Surfayok plasma for 30 s, 1 min, 2 min, 5–8 min. Untreated samples served as controls. All treatments were performed in the scanning mode, as previously described by Loupová et al.^49^. For each condition, two biological replicates were used, each composed of two technical replicates.

Immediately after the treatment, the treated and untreated (control) microbial cells were transferred into suspension. The entire agar plates from two technical replicates (for a given condition) were carefully transferred into 20 ml of sterile PBS in a Falcon tube. These tubes were incubated in a shaking thermostat at 37 °C for 20 min to facilitate detachment of cells. Afterwards, the agars were removed using sterile tweezers, and the suspension was filtered through a 70 μm sterile syringe filter to eliminate residual agar particles. Then, the filtrate was centrifuged at 3000 rpm for 8 min. The supernatant was removed, and the resulting pellet was resuspended in 1 ml PBS and transferred into 1.5 ml Eppendorf tubes. A second centrifugation at the same speed and duration was performed, after which the supernatant was removed, and the cells were resuspended in 500 µl of Millonig’s phosphate buffer. The suspension was washed twice under identical centrifugation conditions. Finally, the cells were fixed in 600 µl of glutaraldehyde and stored at 5 °C for 24 h. After the fixation with glutaraldehyde, a continual dehydration procedure was followed to prepare the samples for SEM and TEM.

#### Scanning electron microscopy

For the scanning electron microscopy, the samples fixed in Millonig phosphate buffered glutaraldehyde (3%) were washed in Millonig buffer, post-fixed in osmium Millonig buffered (OsO_4_ 2%) solution, washed Millonig buffer, dehydrated in 50, 70, 90, and 100% Aceton and dried in HMDS (hexamethyldisilazane, Sigma-Aldrich, Czech Republic). The samples were then put on the carbon tabs attached on the aluminium holder and platinum/palladium coated (Cressington sputter coater 208 h, UK). The samples were observed under scanning electron microscope Hitachi SU 8010 (Hitachi High Technologies, Ltd., Japan) at magnification of 4000–35,000× (at 10 kV, SE detector, working distance wd 8 mm).

#### Transmission electron microscopy

Similarly for the ultrathin section the samples fixed in 3% glutaraldehyde in Millonig phosphate buffer were washed in Millonig buffer, post-fixed in 2% OsO_4_ solution in Millonig phosphate buffer, dehydrated in 50, 70, 90, and 100% aceton and embedded in Epon-Durcupan mixture (Epon 812 Serva, Germany; Durcupan, ACM Fluka, Switzerland). The ultra-thin sections in 70 nm were sectioned by ultramicrotom Leica UC 7, Austria. Then were contrasted with 2% uranyl acetate and 2% lead citrate and observed at 80 kV under EM Philips 208 S Morgagni (FEI, Czech Republic) transmission electron microscope. More details on the preparation of samples for electron microscopy can be found in the study by Kulich et al.^70^

## Electronic Supplementary Material

Below is the link to the electronic supplementary material.

Supplementary Material 1

## Data Availability

The datasets generated and/or analysed during the study are available from the corresponding author on reasonable request.

## Abbreviations

ATP: Adenosine triphosphate
*C. acnes*: *Cutibacterium acnes*
CAP: Cold atmospheric-pressure plasma
CFU: Ccolony forming unit
DBD: Dielectric barrier discharge
DNA: Deoxyribonucleic acid
*E. coli*: *Escherichia coli*
EV: Extracellular vesicle
MW: Microwave
*N. glabratus*: *Nakaseomyces glabratus*
OES: Optical emission spectroscopy
PBS: Phosphate buffered saline
PDV: Peroxisome-derived vesicle
RF: Radio frequency
RNS: Reactive nitrogen species
ROS: Reactive oxygen species
RONS: Reactive oxygen and nitrogen species
*S. epidermidis*: *Staphylococcus epidermidis*
SEM: Scanning electron microscopy
TEM: Transmission electron microscopy
UV: Ultraviolet radiation
