# Correction: Cold microwave plasma jets for wound healing: antimicrobial efficacy, mechanisms and changes in microbial cells

**DOI:** 10.1038/s41598-026-57966-5

**Published:** 2026-06-18

**Authors:** Kristína Trebulová, Veronika Loupová, Barbora Chobotská, Lukáš Kletzander, Přemysl Menčík, Zdenka Kozáková, Jan Hrudka, Joanna Pawlat, Pavel Kulich, František Krčma

**Affiliations:** 1 Faculty of Chemistry, Brno University of Technology, Purkyňova 118, 612 00 Brno, Czech Republic; 2 University of Chemistry and Technology Prague, Technická 5, Dejvice, 166 28 Praha 6, Czech Republic; 3 Veterinary Research Institute, Hudcova 296/70, 621 00 Brno, Czech Republic; 4 Department of Electrical Engineering and Smart Technologies, Lublin University of Technology, Nadbystrzycka Street 38A, 20-618 Lublin, Poland

Correction to: *Scientific Reports* 10.1038/s41598-026-42650-5, published online 06 March 2026

In the original version of this article caption of Figure 11 was given as caption of Figure 3. Therefore, all the subsequent figure captions were incorrectly typeset.

The original Article has been corrected.

