# Supplementary material for: Cold microwave plasma jets for wound healing: antimicrobial efficacy, mechanisms and changes in microbial cells
Source: Sci Rep. 2026 Mar 6;16:12339. doi: 10.1038/s41598-026-42650-5 (PMC13079806; doi:10.1038/s41598-026-42650-5)
Supplement: Supplementary file 1 — Supplementary Material 1 [file 41598_2026_42650_MOESM1_ESM.docx]

**Supplementary information**

*Table 2: Calibration equations for the tested microorganisms*

| microorganism | calibration equation |  |
| --- | --- | --- |
| *E. coli* | *y* = 2.853*x* | *where for y*= 1*, x*= 10^9^ CFU/ml |
| *S. epidermidis* | *y* = 2.186*x* |  |
| *C. acnes* | *y* = 1.76*x* |  |
| *N. glabratus* | *y* = 3.475*x* | *where for y*= 1*, x*= 10^7^ CFU/ml |

*Table 3: Summary of inhibited area sizes for all studied microorganisms and two tested discharges*

| Microorganism | Discharge | Treatment time [s] | Inhibited area [cm^2^] | St. Deviation [cm^2^] |
| --- | --- | --- | --- | --- |
| *E. coli* | SY | 30 | 3.58 | 0.68 |
|  |  | 60 | 7.68 | 0.95 |
|  |  | 120 | 8.29 | 0.12 |
|  |  | 300 | 11.39 | 1.10 |
|  | SW | 30 | 3.07 | 0.74 |
|  |  | 60 | 6.22 | 0.58 |
|  |  | 120 | 7.75 | 0.96 |
|  |  | 300 | 8.84 | 0.72 |
| *S. epidermidis* | SY | 30 | 3.39 | 0.32 |
|  |  | 60 | 5.02 | 0.30 |
|  |  | 120 | 6.93 | 0.48 |
|  |  | 300 | 8.44 | 0.58 |
|  | SW | 30 | 2.21 | 0.41 |
|  |  | 60 | 5.58 | 0.18 |
|  |  | 120 | 6.67 | 0.17 |
|  |  | 300 | 8.00 | 0.55 |
| *C. acnes* | SY | 30 | 4.45 | 0.38 |
|  |  | 60 | 5.34 | 0.76 |
|  |  | 120 | 6.60 | 0.52 |
|  |  | 300 | 8.07 | 0.38 |
|  | SW | 30 | 2.17 | 0.43 |
|  |  | 60 | 3.50 | 0.53 |
|  |  | 120 | 4.93 | 0.21 |
|  |  | 300 | 7.18 | 0.97 |
| *N. glabratus* | SY | 30 | 4.35 | 0.36 |
|  |  | 60 | 5.67 | 0.45 |
|  |  | 120 | 6.87 | 0.47 |
|  |  | 300 | 8.82 | 0.64 |
|  | SW | 30 | 1.61 | 0.18 |
|  |  | 60 | 2.50 | 0.28 |
|  |  | 120 | 3.96 | 0.41 |
|  |  | 300 | 4.72 | 0.43 |
